# GmWRKY49, a Salt-Responsive Nuclear Protein, Improved Root Length and Governed Better Salinity Tolerance in Transgenic *Arabidopsis*

**DOI:** 10.3389/fpls.2018.00809

**Published:** 2018-06-26

**Authors:** Zhaolong Xu, Qasim Raza, Ling Xu, Xiaolan He, Yihong Huang, Jinxin Yi, Dayong Zhang, Hong-Bo Shao, Hongxiang Ma, Zulfiqar Ali

**Affiliations:** ^1^Salt-soil Agricultural Center, Institute of Agricultural Resources and Environment, Jiangsu Academy of Agricultural Sciences, Nanjing, China; ^2^Department of Plant Breeding and Genetics, Muhammad Nawaz Shareef University of Agriculture, Multan, Pakistan; ^3^Rice Research Institute, Kala Shah Kaku, Pakistan; ^4^Jiangsu Key Laboratory for Bioresources of Saline Soils, Jiangsu Synthetic Innovation Center for Coastal Bio-agriculture, Yancheng Teachers University, Yancheng, China; ^5^Institute of Grain Crops, Jiangsu Academy of Agricultural Sciences, Nanjing, China

**Keywords:** *Glycine max*, *GmWRKY49*, overexpression, salinity tolerance, WRKY TFs

## Abstract

Plant WRKY transcription factors (TFs) are active guardians against pathogens’ insurgency, key components in developmental processes, contributors in signal transduction pathways, and regulators of diverse biotic and abiotic stress responses. In this research, we isolated, cloned, and functionally characterized a new WRKY TF GmWRKY49 from soybean. GmWRKY49 is a nuclear protein which contains two highly conserved WRKY domains and a C_2_H_2_-type zinc-finger structure. The normalized expression (log_2_ ratio) of *GmWRKY49* was 2.75- and 1.90-fold in salt-tolerant and salt-susceptible soybean genotypes, respectively. The transcripts of *GmWRKY49* could be detected in roots, stems, leaves, flowers, and almost no expression in pod tissues. The salinity-tolerance response of this gene was studied through overexpression in soybean composite seedlings and transgenic *Arabidopsis*. The effect of *GmWRKY49* overexpression on root length of transgenic *Arabidopsis* was also investigated. Under salt stress, several parameters including germination rate, survival rate, root length, rosette diameter, relative electrolyte leakage, and proline content were significantly higher in composite seedlings and transgenic *Arabidopsis* than those in wild-type. Moreover, *GmWRKY49* enhanced salinity tolerance in soybean mosaic seedlings and transgenic *Arabidopsis*. These results suggest that *GmWRKY49* is a positive regulator of salinity tolerance in soybean and has high potential utilization for crop improvement.

## Introduction

Being sessile, plants are forced to cope with their immediate environment and encounter multiple biotic and abiotic stresses simultaneously. Extreme environmental conditions impact detrimental effects on the growth and development of economically important crops. Soil salinity is a major abiotic stress worldwide (Rengasamy, 2006), and continued salinization of agricultural land threatens crop productivity, particularly in irrigated land areas (Munns and Tester, 2008). To ensure future food security, improving the salt-tolerance of conventional crops may be a significant contribution toward stabilizing the productivity of the crops in salt-affected cultivatable soils.

Plants cope with adverse climatic conditions by regulating the expression of stress-responsive genes. Stress-responsive genes are categorized into structural or regulatory protein-encoding genes, including transcription factors (TFs), and signal-related protein kinases-encoding genes (Hirayama and Shinozaki, 2010). Several stress-responsive TF families such as WRKY, bZIPs, AP2/ERF, DREB, MYB, and NAC are reported to be involved in biotic and abiotic stress responses (Chen et al., 2007; Liao et al., 2008; Nuruzzaman et al., 2013; Llorca et al., 2014; Phukan et al., 2016; Gu et al., 2017). These TFs bind to specific *cis*-acting elements in the promoter region (Dolfini et al., 2012) and modulate the expression of several stress-responsive genes. Some TFs have been recently engineered to enhance salt-tolerance (Xu et al., 2016; Hichri et al., 2017; Li et al., 2017).

WRKY family is one of the largest TF families and extended throughout the green lineage but absent in animals. Among all stress-responsive TF families, WRKYs are of special interest, as they regulate diverse plant responses and processes (Ülker and Somssich, 2004). All WRKY proteins contain at least one DNA binding domain (DBD) and a zinc-finger motif, which can be either C_2_H_2_-type (Cx_4_-_5_Cx_22_-_23_HxH) or C_2_HC-type (Cx_7_Cx_23_HxC). Additionally, they contain a nuclear localization signal (NLS), a kinase domain, a TIR-NBS-LRR domain, leucine zippers, Ser/Thr-rich stretches, and Gln- and Pro-rich regions (Chen et al., 2012), inferring diverse functions under multifarious signaling cascades. The N and C termini of that WRKY domain contain highly conserved WRKYGQK and Zn-finger motifs, respectively; both are necessary for DNA binding activity. Mutations in invariable WRKYGQK motif significantly reduce the binding activity and substitutions of conserved Cysteine and Histidine Zn-finger residues can abolish the binding activity (Maeo et al., 2001). The WRKY DBD generally interacts with W-box core motif TTGACC/T and other clustered W-boxes present in the promoter region of target genes. The DBD also contains four β sheets (β_1_–β_4_). β_1_ and β_2_ are highly conserved, whereas β_3_ and β_4_ show discrepancy in terms of amino acid numbers and conservation. Depending on the number of WRKY domains and type of zinc-finger motifs, all WRKY TFs are categorized into three groups. Group I proteins contain two WRKY domains and a C_2_H_2_-type zinc-finger structure. Groups II and III are marked by only one WRKY domain with C_2_H_2_ and C_2_HC-type zinc-finger structures, respectively. Group II proteins are further categorized into five subgroups (IIa–IIe) (Eulgem et al., 2000; Zhang and Wang, 2005; Rushton et al., 2010). WRKY proteins have the capacity to interact with multiple partners through dimers’ formation. In contrast to bZIP TFs that bind only as dimers, WRKYs have more diverse binding mode as they are capable of binding as monomers, dimers, and even as trimers (Xu et al., 2006; Ciolkowski et al., 2008). WRKY TFs regulate diverse plant responses and processes. WRKY proteins contribute in plant defense mechanism against pathogen attacks (Eulgem et al., 1999), are the key components in developmental processes (Johnson et al., 2002), in signal transduction processes (Rushton et al., 2012), and respond to several abiotic stresses simultaneously (Ramamoorthy et al., 2008). *GmWRKY21* transgenic *Arabidopsis* plants were tolerant to cold stress, *GmWRKY54* conferred salt and drought tolerance, while transgenic plants overexpressing *GmWRKY13* showed increased sensitivity to salt and mannitol stress, but decreased sensitivity to abscisic acid (ABA) (Zhou et al., 2008).

Previously, we generated digital gene expression profiling (DGEP) data from a salt tolerant genotype of *Glycine soja* (STGoGS) and a salt-sensitive genotype of *Glycine max* (SSGoGM) in response to salinity stress. Three STGoGS specific and two SSGoGM specific WRKY TFs were expressed and upregulated. Overall, seven common genes were expressed and upregulated in both species (Ali et al., 2012). Of these common genes, the highest differential expression was observed in *GmWRKY49* and *GmWRKY111*; *GmWRKY49* was selected for further molecular characterization. This gene has high sequence similarity with *AtWRKY33* that contains two WRKY domains with a C_2_H_2_-type zinc-finger structure and belongs to group I. *AtWRKY33* and *AtWRKY25* both are NaCl-inducible TFs which enhances salinity tolerance by increasing the sensitivity to ABA (Jiang and Deyholos, 2009). Based on its ortholog function, *GmWRKY49* is assumed to confer salinity tolerance in soybean.

In this study, salt-responsive WRKY TFs were cloned and analyzed in response to NaCl stress. One salt-responsive candidate gene, *GmWRKY49*, was further functionally characterized to explore its role in salinity tolerance. The expression pattern of *GmWRKY49* in soybean seedlings at different time intervals and in specific tissues was inspected under varying salt stress levels. Furthermore, the functional analysis of soybean composite seedlings and transgenic *Arabidopsis* overexpressing candidate gene was also investigated.

## Results

### Phylogenetic and Domain Analyses of GmWRKY TFs and Subcellular Localization of GmWRKY49 Protein

**FIGURE 1:**
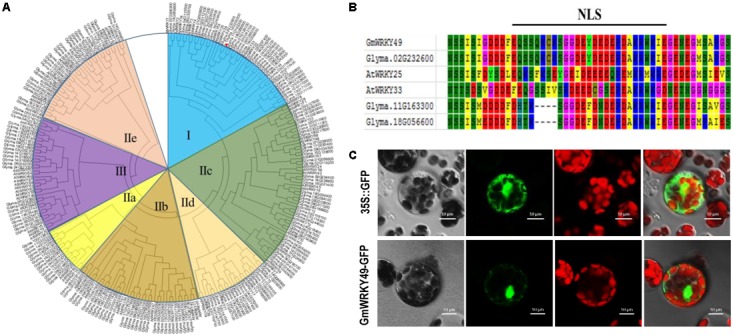
Phylogenetic and domain analyses of WRKYs and subcellular localization of *GmWRKY49* protein. **(A)** Phylogenetic tree inferred after deduced amino acids sequence alignment of 187 *GmWRKYs* and 75 *AtWRKYs*, including *GmWRKY49* (indicated by a red arrow). All WRKY TFs were clustered into three groups named I, II, and III. Second group TFs were further classified into five subgroups (IIa–IIe). *GmWRKY49*, along with *AtWRKY25* and *AtWRKY33*, were clustered into group I. **(B)** Putative nuclear localization signal (NLS); amino acid with different backgrounds shows conserved sequences. **(C)** Subcellular localization of *GmWRKY49* protein in *Arabidopsis* protoplasts. Scale bar = 10 μm.

A comparative phylogenetic analysis of full-length *Arabidopsis* and soybean WRKY TFs, comprising 75 *AtWRKYs* and 187 *GmWRKYs*, classified WRKY genes into three major groups (I, II, and III). The second group was further categorized into five subgroups, named IIa, IIb, IIc, IId, and IIe (**Figure 1A**). The conserved domains and motifs of each group, as based on *AtWRKYs*. Group I contains two WRKY domains with a C_2_H_2_-type zinc-finger motif. Groups II and III contain a single WRKY domain with C_2_H_2_ and C_2_HC-type zinc-finger motifs, respectively, whereas five subgroups (IIa–IIe) differ from each other with respect to amino acids and their conservation. *GmWRKY49* contains two WRKY domains, a C_2_H_2_-type Zn-finger motif and is most similar to *AtWRKY33* and both belong to group I (**Figure 1A**). The same group also contains 12 *Arabidopsis* and 35 soybean WRKY genes. *AtWRKY33* and *AtWRKY25* are NaCl-inducible TFs which respond to multiple abiotic stresses and induction of *AtWRKY33* is dependent upon ABA signaling (Jiang and Deyholos, 2009). The NLS EQSSQKCKSGGDEYDEDEPDAKRWKIE (spanning amino acids 349–375) was also found between two conserved WRKY domains (**Figure 1C**). To detect subcellular localization, a GmWRKY49-green fluorescent protein (GFP) fusion construct and a control construct containing only GFP were created. In both constructs, cauliflower mosaic virus-35S (CaMV 35S)-mediated transcription was driven. By following the PEG-4000-mediated method, constructs were introduced into *Arabidopsis* protoplasts and cells were inspected with a confocal laser scanning microscope. Fluorescence of GmWRKY49-GFP was specifically detected in the nucleus; fluorescence of control construct was distributed throughout the cell (**Figure 1C**). These results indicate that *GmWRKY49* likely functions in the nucleus.

### Sequence Identity of *GmWRKY49* Between Cultivated and Wild Soybean

**FIGURE 2:**
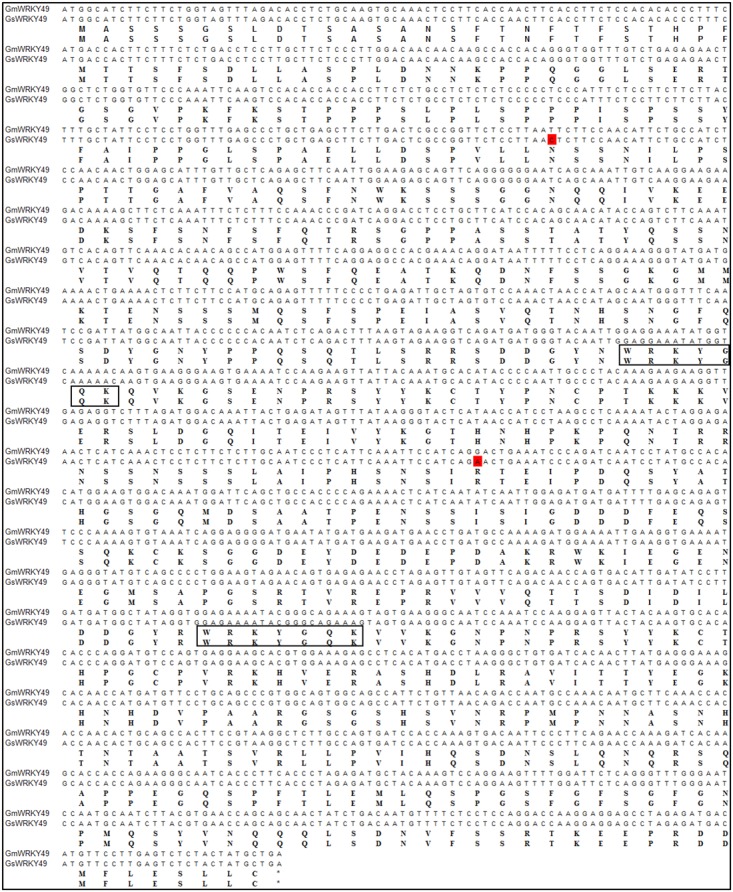
Sequence similarity between cultivated and wild species-specific *GmWRKY49.* Full-length nucleotide and amino acid sequence alignments of *Glycine max* (GmWRKY49) and *Glycine soja* (GsWRKY49) specific genes. Substitutions are highlighted with red boxes. Rectangles encircle WRKY domains and the asterisk symbol indicates termination codon.

For the confirmation of nucleotide sequence similarity, full-length gene primers were designed for *GmWRKY49* and cDNAs from both species were amplified and sequenced. Sequence alignment results indicated that cultivated soybean gene, *GmWRKY49*, has a mutation of two bases in the translated gene sequence (**Figure 2**). The base C at 303 position substitutes with T (303C > T); similarly, base A at 942 position substitutes with G (942A > G). Despite changes in the coding sequence, no changes in amino acid residues were observed. Both mutated codons code for the same amino acids, AAT and AAC code aspartic acid (Asn, N) and AGA and AGG code arginine (Arg, R), due to the degeneracy of genetic code. So, the two mutations are silent mutations, and the structure and functions of *GmWRKY49* protein are conserved between cultivated and wild species.

### Transcriptional Profiles of *GmWRKY49* Under NaCl Treatment

In order to figure out the *GmWRKY49* expression distribution in soybean plant tissues, *in silico* expression of genome-wide GmWRKYs performed, following 14 samples: young leaf, flower, 1 cm pod, pod shells at 10 and 14 days after flowering (DAF), seeds at 10, 14, 21, 25, 28, 35, and 42 DAF, roots, and nodules, and showing that most of WRKY genes expressed in roots, some expressed in flower and young pod, and no expression in seeds, but *GmWRKY49* mainly expressed in roots and flower (**Supplementary Table S1**).

**FIGURE 3:**
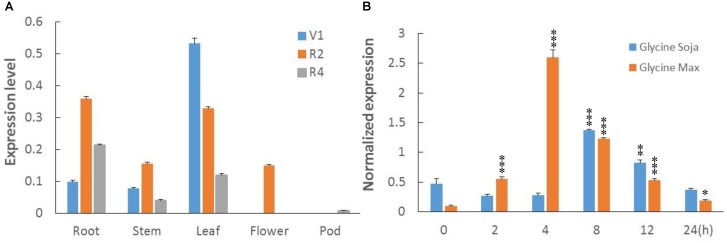
Transcriptional profiles of *GmWRKY49.* **(A)** Tissue-specific transcript abundance of *GmWRKY49* in roots, stems, leaves, flowers, and pods at different developmental stages, the first trifoliate stage (V1), full bloom stage (R2), and full pod stage (R4). **(B)** Expression patterns of *GmWRKY49* in roots of *G. max* and *G. soja* under 0 and 200 mM NaCl stress at different time 0, 2, 4, 8, 12, and 24 h. *P*-values were calculated using Student’s *t*-test and ^∗^*P* < 0.05, ^∗∗^*P* < 0.01, and ^∗∗∗^*P* < 0.001 compared with control and 0 h, respectively.

To clarify transcripts’ abundance in specific tissues, the total RNA was extracted from roots, leaves, stems, flowers, and pods of soybean plants at first trifoliate (V1), full bloom (R2), and full pod (R4) stages. RNA was reverse transcribed to cDNA, and real time-quantitative polymerase chain reaction (RT-qPCR) was performed. Transcription profiles showed relative abundance of *GmWRKY49* transcripts in leaf tissues at the V1 and R2 stages: a decreasing response with growth advance (**Figure 3A**). The expression patterns in roots altered significantly, as minimum transcription observed at the V1, maximum at the R2, and intermediate at the R4 stages. A similar expression pattern was observed in stem tissues. However, *GmWRKY49* was expressed only at the R2 stage in flowers and almost no expression in pod tissues.

Previously, DGEP data were generated from four cDNA libraries of control and NaCl-treated samples of STGoGS and SSGoGM to identify differentially expressed genes (DEGs) (Ali et al., 2012). Expression of *GmWRKY*s was altered in salt-tolerant and salt-sensitive genotypes (**Supplementary Table S1**). Two genes were upregulated and one gene was downregulated specifically in STGoGS. Two genes, *GmWRKY49* and *GmWRKY111*, were upregulated in both species. In comparison with control treatment, *GmWRKY49* was differentially upregulated by 2.75- and 1.90-fold (normalized expression) in *G. soja* and *G. max*, respectively.

To study the expression of *GmWRKY49*, root samples were collected from STGoGS and SSGoGM at different time intervals after salt stress application. The time-course qPCR results showed that transcription of *GmWRKY49* was initially increased at 2 h, reached a maximum at 4 h and then gradually decreased at 8, 12, and 24 h after salt stress, respectively (**Figure 3B**). The normalized expression of *GmWRKY49* increased by approximately 17- and 9-fold in the roots of *G. max* and *G. soja* after 4 and 8 h of NaCl treatments, respectively.

### Overexpression of *GmWRKY49* in Transgenic *Arabidopsis* Plants and Soybean Mosaic Seedlings

**FIGURE 4:**
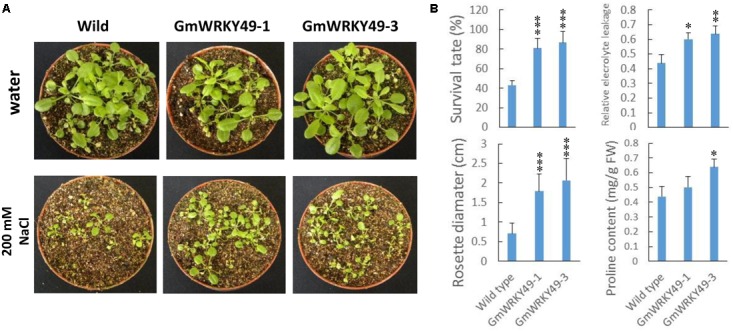
Salinity tolerance of transgenic *Arabidopsis* plants overexpressing *GmWRKY49.* **(A)** Response of WT and *GmWRKY49* transgenic *Arabidopsis* plants under control and saline conditions. **(B)** Survival rate, rosette diameter, relative electrolyte leakage, and proline content differences between WT and transgenic *Arabidopsis* plants in “c” under 200 mM NaCl treatment for 1 week. *P*-values were calculated using Student’s *t*-test and ^∗^*P* < 0.05, ^∗∗^*P* < 0.01, and ^∗∗∗^*P* < 0.001 compared with WT.

In order to study salinity response differences between *GmWRKY49* carrying transgenic *Arabidopsis* lines (*GmWRKY49-1*, *GmWRKY49-3*) and WT, survival rate, rosette diameter, relative electrolyte leakage, and proline contents were determined. Under normal conditions, both transgenic lines showed no significant survival rate difference with WT plants, one exception that, transgenic line *GmWRKY49-1* seedlings showed a weaker growth. As *GmWRKY49* expression was induced by higher salt concentrations, 4-week-old transgenic *Arabidopsis* seedlings were treated with 200 mM sodium chloride for 1 week and wild-type (WT) seedlings were used as a control. Seedlings of *GmWRKY49-1* and *GmWRKY49-3* showed improved growth compared to WT seedlings (**Figure 4A**). Both transgenic lines showed significantly higher survival rate, rosette diameter, relative electrolyte leakage, and proline content values than those of WT (**Figure 4B**). Electrolyte leakage from cell and proline accumulation in the cytoplasm is plant responses to salt stress (Khedr et al., 2003). These results indicate that *GmWRKY49* is an important regulator of salinity tolerance in soybean.

To investigate whether overexpression of *GmWRKY49* in soybean composite seedlings confers salinity tolerance, transgenic seedlings were generated through *Agrobacterium*-mediated transformation and their performance was examined under 200 mM NaCl stress. The results indicated improved salt-tolerance response by soybean mosaic seedlings compared to control seedlings (**Supplementary Figure S1a**). The majority of the seedlings carrying the salt-responsive gene, *GmWRKY49*, were survived with some wilted leaves (**Supplementary Figure S1b**). As expected, more than 91% transgenic seedlings carrying empty vector could not survive and eventually died.

### Effect of *GmWRKY49*-Overexpression on Germination Rate and Root Length of Transgenic *Arabidopsis*

**FIGURE 5:**
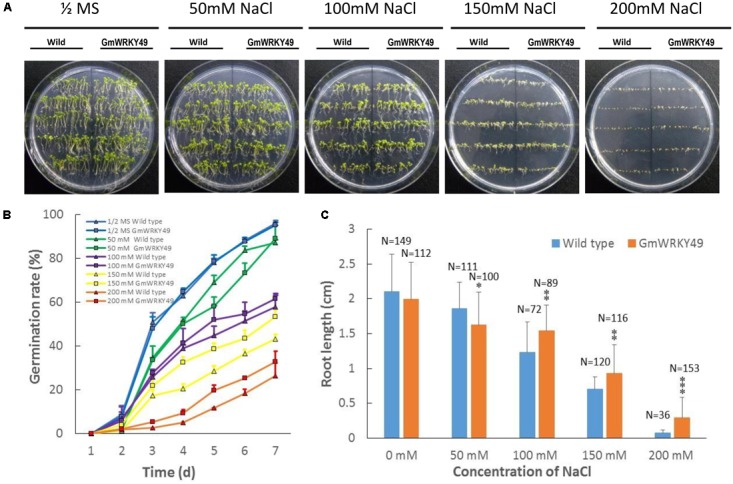
Germination rate and root length of transgenic *Arabidopsis* seedlings overexpressing *GmWRKY49* under different NaCl concentration treatments. Effect of different treatments on germination and root length of wild and transgenic *Arabidopsis* plants overexpressing *GmWRKY49*. **(A,B)** Germination rate differences between WT and transgenic *Arabidopsis* seeds under different treatments. Photographs were taken at the end of experiments, after 7 days. **(C)** Roots’ length was measured after 14 days of 1/2 MS supplemented with 0, 50, 100, 150, and 200 mM NaCl treatments, respectively. *P*-values were calculated using Student’s *t*-test and ^∗^*P* < 0.05, ^∗∗^*P* < 0.01, and ^∗∗∗^*P* < 0.001 compared with wild-type (WT).

To investigate the effect of *GmWRKY49* overexpression on germination and root length, transgenic *Arabidopsis* seedlings were grown on 1/2 strength MS medium supplemented with 0, 50, 100, 150, and 200 mM NaCl treatments. In the absence of salt stress, WT and GmWRKY49 carrying *Arabidopsis* seeds showed non-significant phenotypic and germination rate differences (**Figure 5C**). The germination rate of GmWRKY49 carrying seeds started to decline after 4 days in 50 and 100 mM NaCl treatments; increase in germination rate was observed after 2 and 3 days under 150 and 200 mM treatments, respectively. These results indicate that GmWRKY49 is induced by high salt concentrations. A reduction in root length was observed compared with WT when *GmWRKY49* overexpressing seedlings were under no salt (**Figure 5A**) and 50 mM NaCl stress (**Figure 5C**). However, a significant reduction (*P* < 0.05) in root length was noted when seedlings were under 50 mM stress. At higher salt treatments, 100, 150, and 200 mM, a highly significant (*P* < 0.01) increase in root length was recorded compared to WT. Interestingly, highest gains in root length were observed under 200 mM treatment, confirming that high salt concentrations induce salinity-tolerance response of *GmWRKY49*.

## Discussion

Previous studies have shown that crop plants encounter multiple biotic and abiotic stressors by regulating their stress-related genes. WRKY TFs’ family is one of the largest in higher plants which also plays a pivotal role in biotic and abiotic stress tolerance, particularly in drought and salinity-tolerance (Niu et al., 2012; Xu et al., 2016; Hichri et al., 2017; Liang et al., 2017). In this study, we isolated, identified, and functionally characterized a *G. max* WRKY TF, *GmWRKY49*. Phylogenetic analysis clustered 187 soybean and 75 *Arabidopsis* WRKY family genes into three groups (I, II, and III) and five sub-groups (IIa–IIe) (**Figure 1A**). This clustering of WRKY TFs is similar to those of Rushton et al. (2010) and Song et al. (2016). *GmWRKY49* was clustered with *AtWRKY25, AtWRKY26*, and *AtWRKY33*; these genes belong to WRKY group I. *OsWRKY30* from rice and *TaWRKY2* from wheat also belong to the same WRKY group I (Liang et al., 2017). Similar to other members of WRKY group I, *GmWRKY49* contains two WRKY domains and a C_2_H_2_-type zinc-finger motif. Subcellular localization of the *GmWRKY49-*GFP construct was detected in the nucleus which indicated that it is a nuclear protein (**Figure 1**). Sequence alignment of *GmWRKY49* from wild and cultivated species indicated substitution of two base pairs in the coding region; however, no change in amino acid sequence was observed (**Figure 2**) indicating that it was a silent mutation. Nevertheless, the expression levels of *GmWRKY49* across wild and cultivated species were different (**Figure 3B**). This discrepancy in expression might arise due to the presence/absence of some *cis*-acting elements in the promoter region, as these elements are reported to regulate the gene expression (Dolfini et al., 2012).

The majority of the *GmWRKY* TFs showed differential expression in salt-tolerant and salt-susceptible genotypes of soybean under normal and NaCl stress conditions (**Supplementary Table S2**). The highest expression of *GmWRKY49* was detected in leaves, roots, and nodule tissues (**Figures 3A,B**) which are similar to the transcripts’ abundance of *GmbZIP110*, a NaCl-responsive bZIP TF (Xu et al., 2016). *GmWRKY49* was strongly induced by NaCl stress (**Figure 3B** and **Supplementary Table S1**) in our study, which is consistent with *AtWRKY25* and *AtWRKY33* in *Arabidopsis* (Jiang and Deyholos, 2009). But in a previous study, *GmWRKY49* did not respond to salt stress (Zhou et al., 2008); we infer that the reason is different soybean cultivars used as study materials; “Kefeng No. 1” is more tolerant. *DgWRKY5* in *Chrysanthemum* (Liang et al., 2017) and *TaWRKY2* in wheat (Niu et al., 2012). Composite soybean seedlings carrying candidate gene *GmWRKY49* were more tolerant to salt stress than control seedlings (**Supplementary Figures S1a,b**) which are in accordance with other TFs from WRKY group I: *AtWRKY25*, *AtWRKY33, DgWRKY5*, and *TaWRKY2.* Based on these studies, it is quite possible that TFs of WRKY group I have some analogous effects on abiotic stress tolerance.

High salinity leads to the accumulation of osmotic adjustment substances, such as proline, reactive oxygen species (ROS) and higher electrolyte leakage (de Azevedo Neto et al., 2004). In plants, ROS may injure cytomembrane and cause more oxidative damage of the plasma membrane (Hasegawa et al., 2000). More recently, Liang et al. (2017) measured the high activity of ROS scavenging enzymes (CAT, POD, and SOD) in transgenic *Chrysanthemum* plants compared to WT under control and salt stress conditions. They suggested that *DgWRKY5*, a *Chrysanthemum* salinity-tolerant TF, can regulate the expression of ROS scavengers and weaken the damage of plasma membrane to enhance salinity-tolerance. To withstand salinity stress, plant cells often tend to synthesize and accumulate higher proline content and demonstrate higher relative electrolyte leakage to reduce salinity-induced toxic effects. Proline accumulation is a protective measure taken by the plants to maintain a high osmotic pressure and insurance for moisture absorption from the surrounding environment. It has been reported that plant cells with higher accumulation of organic molecules, especially proline and soluble sugars, had better tolerance to salt stress (Ana et al., 1999). Similarly, higher electrolyte leakage is a hallmark of plant stress-response (Anthony, 1998; Lee and Zhu, 2010) which is detected immediately after stress application and lasts from a few minutes to several hours. This phenomenon is most common in plant roots where leakage of positively charged potassium ions leads to an irreversible K^+^ loss, usually accompanied with higher accumulation of ROS, and often results in programmed cell death under severe stress conditions (Demidchik et al., 2014). In our research, transgenic *Arabidopsis* seedlings overexpressing *GmWRKY49* showed significantly higher relative electrolyte leakage and proline content compared with WT plants (**Figure 4B**). Previously, Xu et al. (2016) also measured significantly higher levels of these and associated them with salt-tolerance response. These results suggest that *GmWRKY49*-overexpressing plants may withstand higher salinity levels by the efficient accumulation of osmotic adjustment substances, enhancing the cation conductance of the plasma membrane and cell membrane protection through the possible regulation of ROS scavenging enzymes.

Salt stress imparts detrimental effects on plant growth and development by reducing germination rate, survival rate, roots length, and fresh weight (Anthony, 1998). However, studies demonstrated that salt-responsive TFs deal with high salt concentrations by maintaining high survival rates and fresh weight, and increasing their roots’ length (Hichri et al., 2017; Li et al., 2017; Liang et al., 2017). In our research, transgenic *Arabidopsis* seeds demonstrated a higher germination rate under 150 and 200 mM NaCl treatments; however, under 50 and 100 mM NaCl treatments, the germination rate of transgenic seeds was lower than WT seeds (**Figure 5B**). This suggests that *GmWRKY49* is induced by high salt concentrations. Furthermore, *GmWRKY49* carrying transgenic seedlings showed significantly higher survival rate and rosette diameter compared to WT after 1 week of 200 mM NaCl stress (**Figure 4B**). Similarly, roots’ length of transgenic *Arabidopsis* seedlings was significantly higher than WT at all NaCl treatments (**Figure 5C**). *Triticum aestivum* TFs, *TaWRKY1, TaWRKY10*, and *TaWRKY33*, also exhibited an increase in germination and survival rates and roots’ length in transgenic *Arabidopsis* and tobacco plants and their overexpression improved drought and salinity-tolerance (Wang et al., 2013; He et al., 2016). These results indicate that *GmWRKY49* may function in plant survival mechanism and root architect improvement under high salinity levels.

## Conclusion

GmWRKY49 is a novel WRKY family TF, which belongs to group I. The differential expression of *GmWRKY49* was detected in salt-tolerant and salt-susceptible genotypes under control and NaCl stress conditions. Transgenic soybean and *Arabidopsis* seedlings overexpressing *GmWRKY49* had better phenotype, survival rate, and salinity-tolerance than WT seedlings. High activity of *GmWRKY49* was observed under more salty conditions which suggest that it might participate in soybean salinity-tolerance response. *GmWRKY49* might directly bind with W-box region and other core elements present in the promoter region and possibly regulate the expression of downstream stress-responsive genes. However, further research efforts might reveal *GmWRKY49* expression at different developmental stages, comprehensive physiological role in salinity tolerance, and possible regulation of stress-related downstream genes.

## Materials and Methods

### Plant Material, Growth Conditions, and Stress Treatments

Previously published procedures were followed for the generation of plant materials, growth conditions, stress treatments, RNA extraction, RT-qPCR, and overexpression analyses (Xu et al., 2016).

The seeds of salt-tolerant and salt-sensitive genotypes of soybean variety were grown under artificially controlled conditions, in pots containing nutritional soil, perlite, and vermiculite at a 1:1:1 ratio. The roots of 3-week-old seedlings were washed with distilled water; each accession was divided into two groups and kept separately into water filled glass beakers. Seedlings of both groups were placed separately into 200 mM NaCl solution and water as a control. For tissue-specific expression analyses, root, leaf, stem, flower, and pod samples were collected from seedlings at first trifoliate (V1), full bloom (R2), and full pod (R4) stages. For total RNA extraction, root samples of both accessions were harvested after 0, 2, 4, 8, 12, and 24 h of NaCl treatment, promptly frozen in liquid nitrogen, and stored at -80°C. RNA was reverse transcribed into first-strand cDNA for further use as a template in PCR. Each experiment contained three biological replicates.

### Identification of Salt-Responsive GmWRKY Genes

Differentially expressed genes, false discovery rate (FDR) < 0.05 (Benjamini and Hochberg, 1995) and fold change log_2_ ratio (stress/normal) either > + 1 or < -1, were identified among control and NaCl-treated samples of STGoGS and SSGoGM by generating DGEP data (Audic and Claverie, 1997). Two WRKY TFs were differentially upregulated across treatments and one, *GmWRKY49*, was selected for further characterization.

### Phylogenetics and *in Silico* Analyses

The peptide sequences of 75 *AtWRKYs* (Eulgem et al., 2000) and 187 *GmWRKYs* (Song et al., 2016) were retrieved from Phytozome v12.1 and aligned using ClustalX2.1^1^. The unrooted phylogenetic tree was constructed using the neighbor-joining method in MEGA version 6 (Tamura et al., 2013).

The expression data for *GmWRKYs* in different plant tissues were mined bioinformatically from SOYBASE^2^, analyzed *in silico*, and displayed using Java-Treeview software.

### Sequence Retrieval, Primer Designing, Amplification, Sequencing, and Domain Analyses of GmWRKY49

CDS and peptide sequences of *GmWRKY49* (Glyma14G200200 and Glyma14g38010) were retrieved from Phytozome v12.1. Forward (5′-ATGGCATCTTCTTCTGGTAGT-3′) and reverse primers (5′-GCATAGTAGAGACTCAAGGAAC-3′) were designed and synthesized by Invitrogen^TM^. cDNA templates from *G. max* and *G. soja* were used for PCR amplification of *GmWRKY49*. Amplicons were cloned and sequenced by GenScript.

Domain analyses were performed using MEME online server^3^. NLS was predicted using online tools at http://nls-mapper.iab.keio.ac.jp/cgi-bin/NLS_Mapper_form.cgi.

### *Arabidopsis* Protoplast Isolation and Sub-cellular Localization

The coding sequence of *GmWRKY49* (1728 bp) was fused to pJIT166-GFP vector driven by CaMV 35S to generate the GmWRKY49-GFP construct. *Arabidopsis* protoplasts were isolated by following the previously published protocol (Yoo et al., 2007). Both fusion (p35S::GmWRKY49-GFP) and control (p35S::GFP) constructs were transformed into *Arabidopsis* protoplast by the PEG4000-mediated method as described by Abel and Theologis (1994). GFP and chloroplast auto-fluorescence’s were measured under a confocal microscope (Zeiss, LSM510 Meta, Carl Zeiss AG) at 480–685 and 495–545 nm, respectively.

### Real-Time Quantitative PCR

For qPCR analysis, total RNAs were extracted from STGoGS and SSGoGM roots under control and 200 mM NaCl treatments. RNA was extracted using the TRIzol^®^ reagent (Invitrogen & Co.) according to the manufacturer’s protocol. Full-length gene-specific forward (5′-TTCCTGCAGCCCGTGCAGT-3′) and reverse (5′-GGCAAGAGCCTTACGGAAGTGGC-3′) primers were used for qPCR and RT-PCR analyses. A soybean *actin* gene (NM_001250673, forward primer, 5′-CGGTGGTTCTATCTTGGCATC-3′; reverse primer, 5′-GTCTTTCGCTTCAATAACCCTA-3′) was used as the internal control. Sample preparation, qPCR, and RT-PCR analyses were performed as Xu et al. (2016) described. Three independent experiments were repeated for qPCR analysis.

### Development of Soybean Composite Seedlings

Seeds of soybean variety “Dongnong 690” were sown in pots containing vermiculite. After cotyledons, emergency, healthy, and robust seedlings were selected for *Agrobacterium*-mediated genetic transformation.

*GmWRKY49* full-length cDNA was cloned into plant expression vector pCXSN under CaMV 35S promoter. The obtained pCXSN-GmWRKY49 construct was transformed into young soybean seedlings at the cotyledonary node and/or hypocotyl via *Agrobacterium rhizogenes*. The seedlings were grown under controlled conditions at 25°C and 60% humidity with 12 h of light/dark cycle. Water was applied as required. After 3 weeks of *Agrobacterium* infection when induced hairy roots could support the plants (Kereszt et al., 2007), the main roots were removed and composite seedlings were transferred into glass tubes containing 1/2 strength Hoagland solution (Hoagland and Arnon, 1950). The glass tubes were placed into a growth chamber set at 25°C/12 h light and 22°C/12 h dark cycle for approximately 1 week. A forward primer that binds to the CaMV 35S promoter region (5′-CAATCCCACTATCCTTCGCAAGACC-3′), a *GmWRKY49* gene-specific reverse primer and genomic DNA from hairy roots as template were used for the PCR-based verification of hairy root transformation. A DNA from pCXSN empty vector carrying composite seedlings was used as a control. The hairy roots of transgenic soybean seedlings were immersed in 1/2 strength Hoagland solution containing 200 mM NaCl and grown for one additional week in the same growth chamber. The Hoagland medium comprising salt was swapped after every other day and data were recorded after 1 week of salt stress. The experiment contained three biological repeats.

### Development of Transgenic *Arabidopsis*

The coding sequence of *GmWRKY49* was cloned into plant expression vector pCXSN under the control of double CaMV 35S promoter, to generate the pCXSN-GmWRKY49 construct. After construct sequence confirmation, the resultant plasmid was transformed into *Agrobacterium tumefaciens* (strain EHA105) to further transform *Arabidopsis* Columbia-0 plants via floral dip method as Xu et al. (2016) described. The obtained transgenic *Arabidopsis* seeds were employed for subsequent experimentation.

For germination assay, 100 seeds of *GmWRKY49* transgenic *Arabidopsis* and WT were surface sterilized and placed on Petri plates containing 1/2 strength Murashige and Skoog (MS) basal medium supplemented with 0, 50, 100, 150, and 200 mM NaCl, respectively. The Petri plates were kept in a growth chamber at 25°C under a 16-h photoperiod for 7 days. For root length measurements, germinated seeds were supplied with the same nutritional and stress treatments for one additional week. The germination rates were recorded everyday after sow for 7 days, whereas the root length was measured after 14 days, separately from each experiment.

For morphological and physiological assays, seeds of transgenic and WT *Arabidopsis* were sown in pots containing vermiculite and nutritional soil (3:1). Pots were placed into the growth chamber set at 22°C/12 h light and 20°C/12 h dark cycle. Water was applied as required. For salt treatment, *Arabidopsis* seedlings after 4 weeks of growth were supplied with 10 ml of 200 mM NaCl every day for 1 week. Untreated seedlings were used as a control. After 1 week of treatment, survival rate, rosette diameter, proline content, and relative electrolyte leakage were measured. The experiment contained three biological repeats.

### Determination of Relative Electrolyte Leakage and Free Proline Content

The relative electrolyte leakage was assayed as described by Cao et al. (2007). Proline accumulation was estimated according to Bates et al. (1973). Each data point represents an average of three independent experiments.

### Statistical Analyses

All data were processed using the SPSS version 15.0 (SPSS, Inc., Chicago, IL, United States). The significance of data was computed by using Student’s *t*-test and *P*-values < 0.05 were considered to be statistically significant. All the error bars were SD (Standard Deviation) value.

## Author Contributions

HM, ZA, and JY designed the experiments. ZX, QR, LX, XH, YH, and DZ finished the experiments. ZX and QR analyzed the data. ZX and QR finished the manuscript. H-BS revised the paper. All authors approved the paper.

## Conflict of Interest Statement

The authors declare that the research was conducted in the absence of any commercial or financial relationships that could be construed as a potential conflict of interest.

## Footnotes

1 http://www.clustal.org/clustal2/

2 http://soybase.org

3 http://meme-suite.org/

## Supplementary Material

FIGURE S1 Salinity-tolerance of soybean composite seedling plants overexpressing GmWRKY49. **(a)** Comparison between the soybean composite seedling carrying an empty vector or a GmWRKY49-overexpression vector. Photographs were taken after 1 week of 200 mM NaCl stress. **(b)** Survival of soybean composite seedlings in “a” under salt stress.

TABLE S1 Genome wide transcript abundance data for full length GmWRKY genes from Soybean.

TABLE S2 Transcript aboundance data of GmWRKY genes from our DGEP data.
