# Supplementary material for: GmWRKY49, a Salt-Responsive Nuclear Protein, Improved Root Length and Governed Better Salinity Tolerance in Transgenic Arabidopsis
Source: Front Plant Sci. 2018 Jun 26;9:809. doi: 10.3389/fpls.2018.00809 (PMC6028721; doi:10.3389/fpls.2018.00809)
Supplement: FIGURE S1 — Salinity-tolerance of soybean composite seedling plants overexpressing GmWRKY49. (a) Comparison between the soybean composite seedling carrying an empty vector or a GmWRKY49-overexpression vector. Photographs were taken after 1 week of 200 mM NaCl stress. (b) Survival of soybean composite seedlings in “a” under salt stress. [file Image_1.PDF]

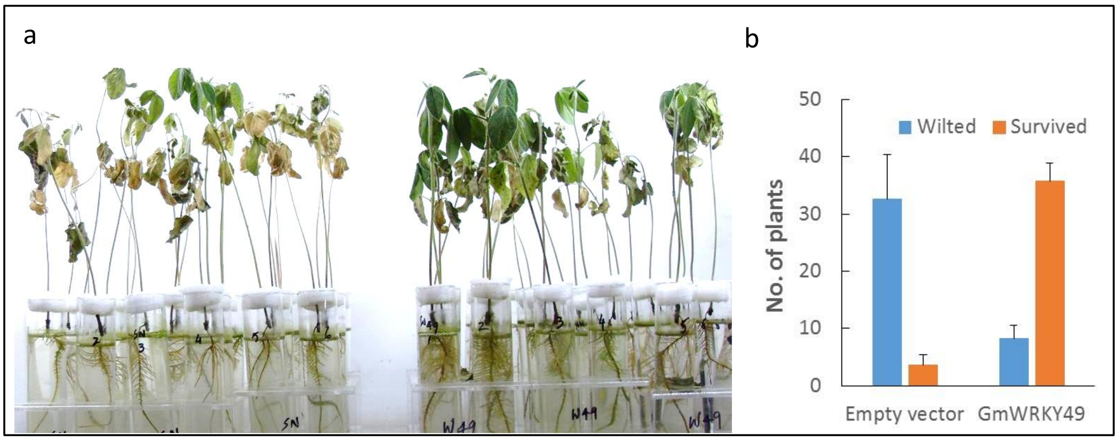

Figure S1: Salinity tolerance of soybean composite seedlings plants overexpressing GmWRKY49

(a) Comparison between soybean composite seedling carrying an empty vector or a GmWRKY49-overexpression vector. Photographs were taken after one week of 200 mM NaCl stress. (b) Survival of soybean composite seedlings in “a” under salt stress.
